# Implementing integrated hypertension and diabetes management using the World Health Organization’s HEARTS model: protocol for a pilot study in the Guatemalan national primary care system

**DOI:** 10.1186/s43058-023-00539-8

**Published:** 2024-01-09

**Authors:** Irmgardt Alicia Wellmann, Luis Fernando Ayala, José Javier Rodríguez, Timothy C. Guetterman, Vilma Irazola, Eduardo Palacios, Mark D. Huffman, Peter Rohloff, Michele Heisler, Manuel Ramírez-Zea, David Flood

**Affiliations:** 1 Research Center for Prevention of Chronic Diseases, Institute of Nutrition of Central America and Panama, Guatemala City, Guatemala; 2 Department of Family Medicine, University of Michigan, Ann Arbor, MI USA; 3 Institute for Clinical Effectiveness and Health Policy, Buenos Aires, Argentina; 4 National Program for the Prevention of Chronic Non-Communicable Diseases and Cancer, Ministry of Health, Guatemala City, Guatemala; 5 Department of Medicine and Global Health Center, Washington University in St. Louis, St. Louis, MO USA; 6 Department of Preventive Medicine, Northwestern University Feinberg School of Medicine, Chicago, IL USA; 7 The George Institute for Global Health, University of New South Wales, Sydney, Australia; 8 Center for Indigenous Health Research, Wuqu’ Kawoq, Tecpán, Guatemala; 9 Division of Global Health Equity, Brigham and Women’s Hospital, Boston, MA USA; 10 Department of Internal Medicine, University of Michigan, Ann Arbor, MI 48109 USA; 11 Veterans Affairs Ann Arbor Center for Clinical Management Research, Ann Arbor, MI USA

**Keywords:** Implementation research, Health policy and systems research, Global health, Hypertension, Diabetes, Guatemala, WHO HEARTS technical package

## Abstract

**Background:**

The HEARTS technical package was developed by the World Health Organization to address the implementation gap in cardiovascular disease prevention in low- and middle-income countries. Guatemala is a middle-income country that is currently implementing HEARTS. National authorities in Guatemala are interested in exploring how hypertension and diabetes management can be integrated in HEARTS implementation. The objective of this study is to conduct a feasibility and acceptability pilot trial of integrated hypertension and diabetes management based on HEARTS in the publicly funded primary care system in Guatemala.

**Methods:**

A single-arm pilot trial for 6 months will be carried out in 11 Ministry of Health primary care facilities starting in September 2023. A planned sample of 100 adult patients diagnosed with diabetes (*n* = 45), hypertension (*n* = 45), or both (*n* = 10) will be enrolled. The intervention will consist of HEARTS-aligned components: Training health workers on *h*ealthy-lifestyle counseling and *e*vidence-based treatment protocols, strengthening *a*ccess to medications and diagnostics, training on *r*isk-based cardiovascular disease management, *t*eam-based care and task sharing, and *s*ystems monitoring and feedback, including implementation of a facility-based electronic monitoring tool at the individual level. Co-primary outcomes of feasibility and acceptability will be assessed using an explanatory sequential mixed-methods design. Secondary outcomes include clinical effectiveness (treatment with medication, glycemic control, and blood pressure control), key implementation outcomes (adoption, fidelity, usability, and sustainability), and patient-reported outcome measures (diabetes distress, disability, and treatment burden). Using an implementation mapping approach, a Technical Advisory Committee will develop implementation strategies for subsequent scale-up planning.

**Discussion:**

This trial will produce evidence on implementing HEARTS-aligned hypertension and diabetes care in the MOH primary care system in Guatemala. Results also will inform future HEARTS projects in Guatemala and other low- and middle-income countries.

**Trial registration:**

ClinicalTrials.gov ID NCT06080451. The trial was prospectively registered on October 12, 2023.

**Supplementary Information:**

The online version contains supplementary material available at 10.1186/s43058-023-00539-8.

Contributions to the literature

- The World Health Organization developed the HEARTS technical package to improve implementation of evidence-based interventions to prevent cardiovascular disease in primary care facilities in low- and middle-income countries. Most HEARTS implementation projects to date have focused on a single risk factor, hypertension.
- This pilot study will investigate how integrated hypertension and diabetes management based on HEARTS can be implemented in the Guatemalan national primary care system.
- Implementation strategies will be developed and selected using an implementation mapping approach. These implementation strategies will inform future HEARTS scale-up projects in Guatemala and other low- and middle-income countries.

## Background

Approximately, 80% of the global burden of hypertension and diabetes occurs in low- and middle-income countries [1]. Widespread adoption of evidence-based treatment of these conditions in high-income countries contributes to markedly better cardiovascular disease (CVD) outcomes than in low- and middle-income countries, where adoption is often limited [2–4]. To address this implementation gap, the World Health Organization (WHO) developed the HEARTS technical package for CVD Management in Primary Health Care (henceforth, “HEARTS”) [5]. HEARTS is a package of evidence-based interventions that align with the US Kaiser Permanente hypertension program [6] and the Pan American Health Organization (PAHO) Standardized Hypertension Treatment Project [7, 8]. The package has six multilevel intervention components forming the acronym “HEARTS”: healthy lifestyle counseling, evidence-based protocols, access to medicines, risk-based management, team care and task sharing, and systems monitoring.

HEARTS is intended to improve CVD prevention within national primary care systems by addressing multiple CVD risk factors. To date, however, HEARTS implementation projects have focused on hypertension as it is the highest-burden risk factor [9, 10]. To further its impact, HEARTS can be expanded to integrate management of other CVD risk factors such as diabetes [11]. As the diabetes-specific HEARTS module (HEARTS-D) primarily focuses on clinical diabetes care, there is a need for generalizable evidence on implementing integrated hypertension and diabetes with the HEARTS framework [11].

Guatemala is a middle-income country with the highest burden of cardiometabolic diseases in Central America [12]. An estimated 32.2% [3] and 13.1% [13] of Guatemalan adults have hypertension and diabetes, respectively, and the two conditions account for one-quarter of national deaths [12]. This project builds on prior hypertension control projects in Guatemala by study investigators, local collaborating organizations, and stakeholders in the Ministry of Health (MOH) and PAHO. From 2017 to 2022, study team members implemented a HEARTS-aligned multicomponent, multilevel hypertension project across MOH primary care facilities in 5 of the country’s 22 departments [14]. In 2021, study team members initiated a HEARTS pilot in 6 MOH primary care facilities. Finally, in November 2022, the Guatemalan MOH officially launched HEARTS. While HEARTS in Guatemala initially focuses on hypertension, national authorities are interested to exploring how diabetes can be integrated into ongoing implementation efforts.

The primary objective of this pilot study is to test the feasibility and acceptability of an integrated model of hypertension and diabetes management based on HEARTS in the publicly funded primary care system in Guatemala. Secondary objectives of this study are to rehearse study procedures and to engage with key stakeholders to develop implementation strategies for a subsequent scale-up project.

## Methods/design

This protocol follows the SPIRIT guidelines for clinical trial protocols (Additional file 1) [15]. Additional files 2 and 3 include the CONSORT [16] and TIDieR [17] checklists. We also applied, as appropriate, guidance on reporting non-randomized pilot studies, conducting pilot implementation studies, and applying mixed-methods to pilot studies [18–20]. Table 1 defines important concepts as used in this study protocol.

**Table 1:** Definitions of important concepts as used in this study protocol

• The term *pilot study* is used in this protocol because the study objective is not only to investigate whether HEARTS can be implemented in Guatemala but also to evaluate a smaller-scale version of a future planned scale-up study [21]
• The term *feasibility* can mean different things in pilot studies [22] and in global cardiovascular disease research [23]. In this protocol, we use *feasibility* to refer to implementation outcomes
• The *evidence-based intervention* (“the thing” [24]) in this study is the HEARTS package of evidence-based interventions. In this protocol, we use the name “Integrated Hypertension and Diabetes Primary Care Model” to refer to this evidence-based intervention as implemented in Guatemala
• This study will not evaluate *implementation strategies* (“the stuff we do to try to help people and places to do ‘the thing’” [24]). However, a key secondary objective of this study is to develop implementation strategies for each intervention component, thus informing a subsequent scale-up study of HEARTS implementation in Guatemala

### Study design

A single-arm pilot trial over 6-month duration will be carried out in starting in September 2023. A single-arm design was chosen as most appropriate to evaluate feasibility and acceptability and to align with recommendations for pilot projects in the HEARTS implementation guide [25].

### Study setting

#### Participating health facilities

**Fig. 1:**
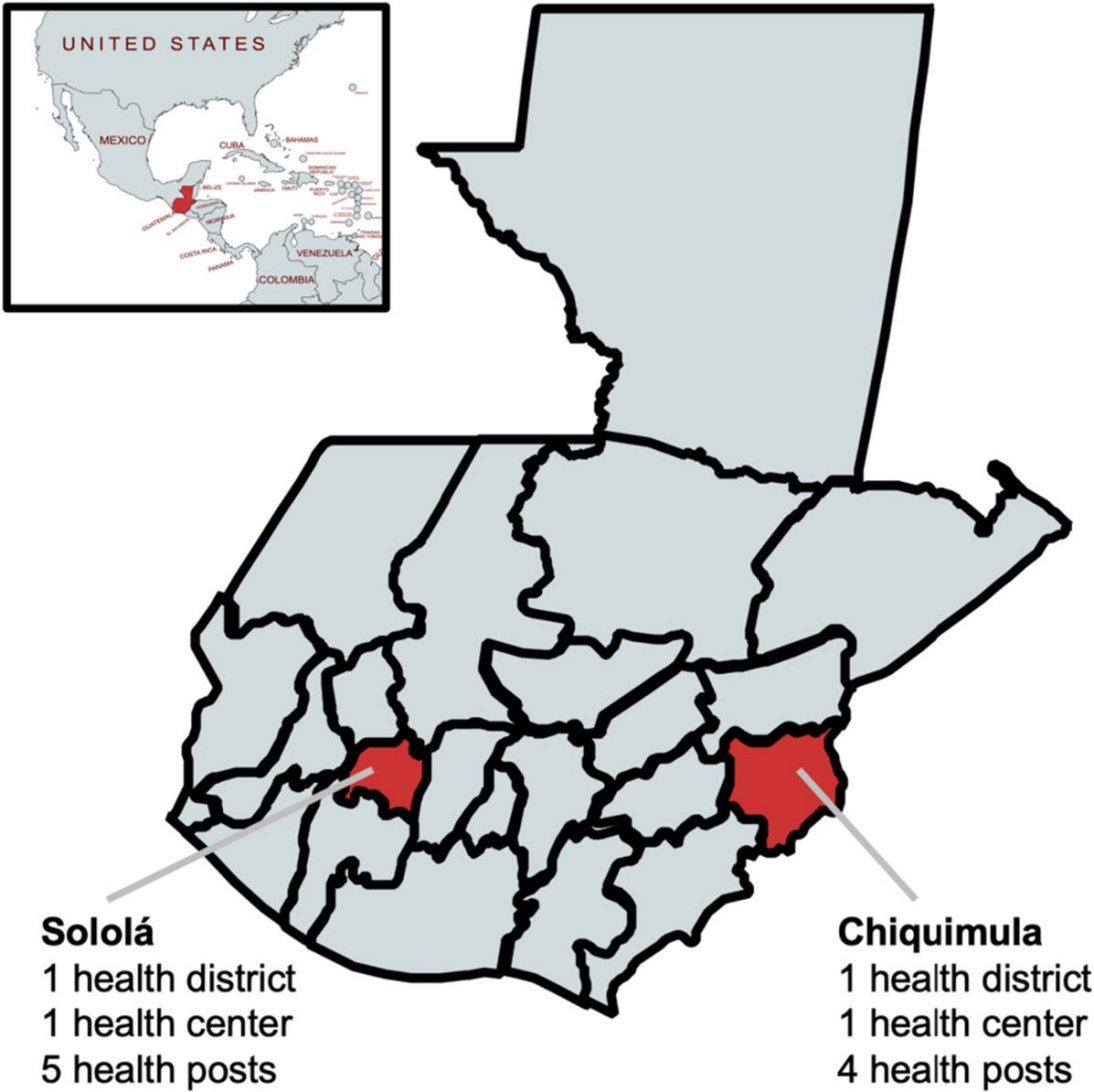
Map of study setting. The pilot study will be carried out in 11 primary care facilities (either health posts or health centers) in two health districts in the Ministry of Health primary care system in Guatemala. Map inset depicts the location of Guatemala relative to neighboring countries

This study will be carried out in 11 MOH primary care facilities in 2 health districts (Fig. 1). The two health districts were selected in consultation with the MOH and PAHO. Each health district includes one second-level primary health facility (health center) and referring first-level primary health facilities (health posts). Both health districts were sites where the study team previously implemented HEARTS-aligned hypertension control projects. The health districts were purposefully selected to represent important areas of diversity in Guatemala across location and ethnicity. Neither health district was part of the initial wave of HEARTS implementation in the MOH. It was also important that each site had motivated MOH leadership [25]. The selected health district in Sololá is in the Central Highlands and has a primarily indigenous Maya population. The selected health district in Chiquimula is in eastern Guatemala and has a primarily non-Indigenous population. Both health districts have poverty rates of 60–70% with large rural populations [26, 27].

#### Study context

##### Health system structure

Approximately, 75% of the population in Guatemala is uninsured [28]. The majority of uninsured patients with hypertension and diabetes in Guatemala depend on the MOH-led system for health care. The MOH system is a national, publicly funded system consisting of multiple levels [28]. The first two levels are the primary care levels where this project will be conducted (health posts and health centers). Health posts are in rural villages, are typically open during business hours on weekdays, and are staffed by 1–2 auxiliary nurses. Auxiliary nurses are full-time MOH employees and have similar training to nursing assistants in the US healthcare system. Their scope of practice includes a wide range of basic preventative and curative primary care services, but auxiliary nurses typically do not provide pharmacological management of noncommunicable diseases such as diabetes or hypertension. Health centers are in urban or semi-urban areas in midsized towns, are open 24/7 for emergencies, and are staffed by professional nurses, general physicians, physicians in training, or a combination thereof. Health centers manage uncomplicated diabetes or hypertension cases. Available resources typically include oral medications and tools for measuring blood glucose and blood pressure. Patients needing insulin therapy, acute inpatient care, or specialist management of diabetes or hypertension complications are referred from health centers to regional or national hospitals.

##### Clinical guidelines

The National Program for the Prevention of Chronic Non-Communicable Diseases and Cancer coordinates hypertension and diabetes policies and guidelines in the MOH [29]. The MOH regularly releases clinical guidelines for primary care clinicians in Guatemala. The most recent hypertension and diabetes guidelines, released in 2018, were updated in 2023 [30] and are generally consistent with international guidelines [31]. The main challenge relating to clinical guidelines in Guatemala is the need for investments to support guideline implementation, including staffing, training and supervision, and equipping primary care facilities with clinical resources.

##### Clinical data systems

At present, there is no standardized paper or electronic patient medical record in the MOH-led health system. As a result, there is difficulty tracking individual patients over time or between health system levels. There is also no official diabetes or hypertension registry. The MOH has an electronic tool, the Health Management Information System, which primarily serves to monitor resource utilization, especially medications dispensed (*Sistema de Información Gerencial de Salud* [SIGSA]). The SIGSA system is not designed to capture longitudinal patient data, and thus, in practice, clinicians cannot use the system to provide clinical care with information stored during prior clinical visits.

##### Availability and cost of medications and diagnostics

Guatemalan laws guarantee that health care including medications is free of charge at MOH health facilities [28]. The MOH thus is responsible for ensuring the availability of quality medications and supplies relating to hypertension and diabetes. At the primary care level, the most commonly available medications for hypertension are hydrochlorothiazide, enalapril, and losartan; the most commonly available medications for diabetes are metformin and glimepiride. Tests such as hemoglobin A1c (HbA1c), creatinine, or cholesterol are not available at MOH-led primary care facilities, though patients sometimes solicit testing at private laboratory facilities. Stockouts of medications and diagnostics occur [32].

#### Context of HEARTS implementation in Guatemala

In November 2022, with support from PAHO, the Guatemalan MOH committed to participate in the “Hearts in the Americas” initiative [33]. The MOH plans a stepped implementation of HEARTS across the country. The first 36 health districts across 6 of 22 departments in the country were enrolled in late 2022 and 2023. (“Departments” are first-level political subdivisions analogous to US states.) Neither of the sites in this pilot was included in the initial wave of HEARTS implementation in Guatemala. The MOH has committed resources to HEARTS, including provision of medications and supplies. To date, HEARTS implementation in Guatemala has focused only on hypertension management at MOH health centers. Diabetes management is not currently part of the MOH’s HEARTS implementation plans.

### Eligibility criteria

#### Patient participants

##### Inclusion criteria

All nonpregnant adults aged ≥ 18 years with diagnoses of type 2 diabetes, hypertension, or both conditions who present for routine care at participating MOH primary health facilities over 6 months will be included (“patient participants”).

Both previously diagnosed and newly diagnosed patients will be eligible. Previously diagnosed patients will be identified by MOH primary care clinicians who take medical histories as part of routine care. Newly diagnosed patients will be identified by MOH primary care clinicians who apply hypertension and diabetes diagnostic criteria from national guidelines [30, 31]. Diabetes diagnostic criteria for newly diagnosed patients will be fasting glucose ≥ 126 md/dl, 2-h postprandial glucose ≥ 200 md/dl, or *HbA1c* ≥ 6.5%. Hypertension diagnostic criteria for newly diagnosed patients will include systolic blood pressure ≥ 130 mmHg or diastolic blood pressure ≥ 80 mmHg; a new hypertension diagnosis must be based on the average of at least two measurements performed on two separate occasions.

##### Exclusion criteria

Participants with confirmed or suspected type 1 diabetes or who are pregnant will be excluded, as these patients are not managed at MOH health centers or health posts. Participants with a prior history of CVD will not be excluded.

#### Other participants

All MOH staff (i.e., physicians, nurses, and auxiliary nurses) and stakeholders on the Technical Advisory Committee will be eligible for participation in the implementation assessment of the pilot (“MOH participants”).

### Intervention

**Fig. 2:**
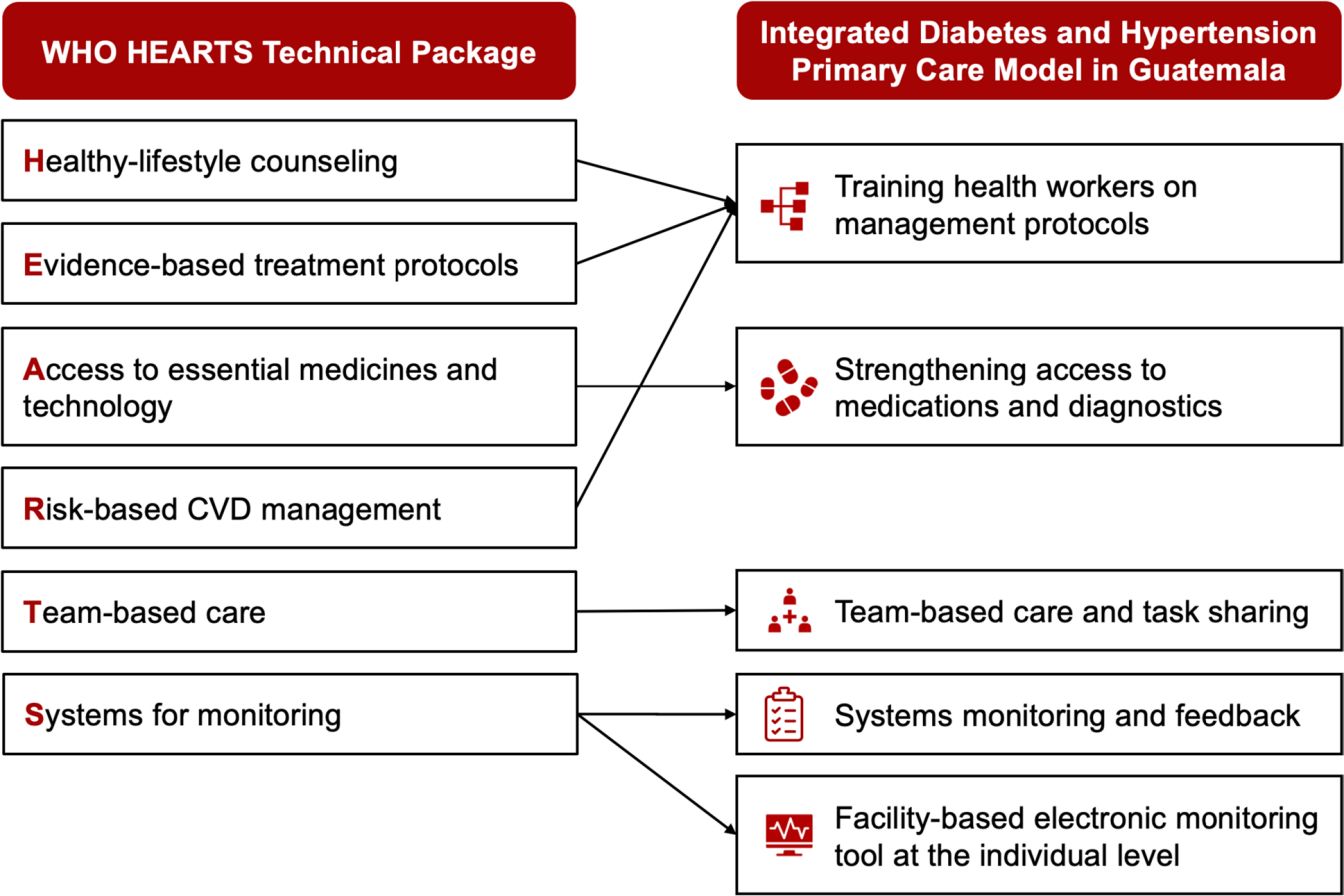
Intervention components. The intervention piloted in this study (“Integrated Hypertension and Diabetes Primary Care Model” [*Modelo Integral de Hipertensión y Diabetes en la Atención Primaria*]) consists of five HEARTS-aligned components that together comprise the package of evidence-based interventions implemented in this pilot study

The intervention piloted in this study (“Integrated Hypertension and Diabetes Primary Care Model” [*Modelo Integral de Hipertensión y Diabetes en la Atención Primaria*]) consists of five HEARTS-aligned components that together comprise the package of evidence-based interventions that we seek to implement (Table 1 and Fig. 2). These components were selected, adapted, and evaluated in our prior hypertension projects in Guatemala [14, 34–37], but the focus on integrated diabetes and hypertension management is novel in this study (Table 1).

#### Training health workers on hypertension and diabetes management

This component includes training on healthy-lifestyle counseling, evidence-based treatment protocols, and risk-based CVD management. Training workshops will be conducted for first- and second-level health workers, including auxiliary nurses, professional nurses, and physicians. The goal is to provide instruction in standardized screening, diagnostic, and treatment protocols for hypertension and diabetes in MOH guidelines [30, 31]. Initial workshops will be divided into two blocks, each lasting 2 days, in the first month of the project. Pre- and post-training assessments will be conducted to assess changes in knowledge. A refresher training session will be provided in the fourth month of the project. Training will be delivered in each health district’s office headquarters. The training will adapt a curriculum previously used in the study team’s HEARTS-aligned projects and will be approved by the Department of Health Training and Education (*Departamento de Promoción y Educación en Salud* [PROEDUSA]), which is the unit in the MOH charged with continuing medical education. Workshop content will include the following topics: an introduction to hypertension and diabetes, diagnostic criteria, use of stepped treatment protocols, treatment goals, medication side effects, counseling to promote lifestyle changes, motivational interviewing, team-based care, capture and use of electronic patient data, and other topics. Participants will have knowledge assessments before and after training workshops. Of note, MOH treatment goals for diabetes are fasting glucose 70–115 mg/dL, postprandial glucose 70–160 mg/dl, or HbA1c < 7.0; MOH treatment goals for hypertension are < 130/80 mm/Hg [31, 38, 39].

#### Team-based care and task sharing

To implement hypertension and diabetes care in health posts, we will implement a team-based, task-sharing care model between auxiliary nurses staffing health posts and prescribing clinicians (i.e., physicians or professional nurses) at health centers. This intervention component was implemented in the study team’s prior hypertension project and has been approved by the MOH [14]. Physicians or professional nurses will make initial patient treatment plans. Auxiliary nurses working in health posts will implement treatment plans by dispensing medications, monitoring glycemic or blood pressure control, and titrating medications under physician supervision. Care coordination meetings will be held in person or remotely at least once per month to review patient registries and make recommendations for patients whose hypertension or diabetes is not adequately controlled according to MOH guidelines [30, 31]. In our prior projects, monthly meetings have been difficult to operationalise [40]. Therefore, we may suggest an alternative approach in which auxiliary nurses at health posts communicate with physicians at health centers in real time via text messages or phone calls to make treatment changes for uncontrolled patients.

#### Strengthening access to medications and diagnostics

We have extensive experience collaborating with the MOH to improve medication procurement and logistics at MOH health centers and health posts. In the study team’s hypertension project in five departments, nearly 100% availability of key medications was achieved in MOH facilities over 3 years. In the current project, we will expand the scope to improve access to diagnostics and medications for diabetes at participating MOH primary care facilities. We will coordinate with and train MOH staff on topics that include forecasting demand, seasonal budgeting, storage, shipping, and other topics. Feedback will be provided to MOH staff based on monthly, in-person health facility assessments of medication availability. The focus will be on a small set of MOH-priority medications and diagnostics. Drugs include antihypertensive medications (i.e., hydrochlorothiazide, enalapril, losartan) and oral hypoglycemic agents (i.e., metformin and glimepiride). Of note, single-pill combination medications recommended in HEARTS are not yet available in the MOH system [41]. Diagnostics include blood pressure cuffs and monitors, glucometers, lancets, and glucose strips. As noted in “Study context,” all medications and diagnostics are provided freely to patients in the MOH. The implementation of a facility-based electronic monitoring tool, described below, also functions to improve the availability of medications and diagnostics by providing enhanced data to monitor supply and demand at primary care facilities.

#### Facility-based electronic monitoring tool at the individual level

The study team previously has collaborated with the MOH to pilot the District Health Information System 2 (DHIS2) in health centers and health posts. DHIS2 is an open-source, facility-based electronic monitoring tool that can monitor key indicators at the individual and aggregate levels [42]. We will implement the DHIS2 system including both hypertension and diabetes modules in MOH primary care facilities. Registries of patients with hypertension and diabetes will be constructed at each MOH facility. The project will provide hardware (e.g., tablets or desktop computers), Internet connectivity, technical support, and training and supervision of MOH staff. The DHIS2 system will be hosted on a centralized server, allowing trained health workers to enter data and monitor patient data in real time. In the WHO classification system for digital health interventions, this intervention component is a healthcare provider intervention focusing on client health records [43].

#### Systems monitoring and feedback of key indicators

The HEARTS component of “Systems for Monitoring” requires the use of routine administrative clinical data to monitor key indicators and to iteratively improve the quality of hypertension and diabetes care (Fig. 2) [44]. Each month, we will present aggregate reports of key indicators using data drawn from DHIS2 to MOH stakeholders at the health district (i.e., municipal) and health area (i.e., departmental) levels. The key indicators will be the same as the HEARTS-aligned secondary outcomes described below. We will use a suite of DHIS2 visualization tools built by PAHO, including maps, graphs, and dashboards. In the WHO classification system for digital health interventions, this intervention component is a health system manager intervention focusing on facility management [43]. Facility-level monitoring using DHIS2 will be complemented by ongoing health worker training and site supervision visits.

### Outcomes

#### Primary outcomes

Primary outcomes will be feasibility and acceptability as defined in the implementation outcomes framework [45]. Table 2 summarizes these outcomes with minimum benchmarks. Feasibility and acceptability will be assessed using integrated quantitative and qualitative data (mixed methods). Given the study team’s prior experience with HEARTS-aligned projects, the focus of the feasibility and acceptability assessments will be on integrating diabetes into the HEARTS model. Feasibility is the extent to which a new intervention can be successfully carried out in an organization [45]. Among MOH participants, feasibility will be assessed through the four-item feasibility of intervention measure (FIM) questionnaire [46] and semi-structured interviews. Among patient participants, feasibility will be assessed using enrollment data. Acceptability is the stakeholders’ perception that a new intervention is agreeable or satisfactory [45]. Among MOH participants, acceptability will be assessed using the acceptability of intervention measure (AIM) questionnaire [46] and semi-structured interviews. Among patient participants, acceptability will be assessed using follow-up visit data and semi-structured interviews.

**Table 2:** Measures of feasibility and acceptability and their benchmarks

Measure	Minimum benchmark
*Feasibility*	
Feasibility questionnaire (FIM) among MOH participants	Median ≥ 3.5^a^
Reasons for perceptions of feasibility/infeasibility	N/A
Number of patient participants with diabetes enrolled per health district^b^	25
Number of patient participants with hypertension enrolled per health district^b^	25
*Acceptability*	
Acceptability questionnaire (AIM) among MOH participants	Median ≥ 3.5^a^
Proportion of patient participants with subsequent follow-up visit within 3 months (among those enrolled with ≥ 3 months remaining in pilot)	75%
Reasons for perceptions of acceptability/infeasibility among patient and MOH participants	N/A
Reasons for dropouts among patient participants	N/A

*Abbreviations*: *AIM*, Acceptability of Intervention Measure, *FIM*, Feasibility of Intervention Measure, *MOH* Ministry of Health

Primary outcomes in this pilot study are feasibility and acceptability as defined in the Implementation Outcomes Framework

^a^The FIM and AIM scales range from 1 to 5 with higher values implying greater feasibility or acceptability, respectively; the participant’s mean score across the four questions will be used

^b^Enrollment is defined by a patient having at least one clinic visit entered in the DHIS2 or equivalent longitudinal medical record system; a given patient may have both diabetes and hypertension and thus count toward each benchmark

#### Secondary outcomes

Secondary outcomes include clinical outcomes, implementation outcomes, and patient-related outcome measures (Table 3). Clinical effectiveness outcomes are based on recommended HEARTS monitoring indicators [44, 47]. Clinical outcomes are assessed to provide pilot data to key MOH stakeholders and to rehearse study procedures rather than to evaluate effectiveness. Implementation outcomes [45] will assess facility-level adoption and the fidelity of implementation of each intervention component. Patient-related outcome measures relating to diabetes will be conducted to explore and refine the study team’s use of these instruments in Guatemalan Spanish and local Mayan languages (Kaqchikel or Tz’utujil). Measures include diabetes distress, quality of life, and self-care assessments.

**Table 3:** Outcomes and data sources

Outcome	Description and data sources
**Primary outcomes**	
Feasibility	FIM questionnaires and MOH data from DHIS2 (quantitative); semi-structured interviews with MOH participants (qualitative)
Acceptability	AIM questionnaires and MOH data from DHIS2 (quantitative); semi-structured interviews with patient and MOH participants (qualitative)
**Secondary outcomes**	
* Clinical outcomes*	
Number of patients receiving hypertension medication treatment per month (“hypertension treatment rate”)	MOH data from SIGSA (quantitative)
Number of patients receiving diabetes medication treatment per month (“diabetes treatment rate”)	MOH data from SIGSA (quantitative)
Proportion achieving glycemic control (*FBG* < 115 mg/dl or *RBG* < 160 mg/dl) among patients with diabetes	MOH data from DHIS2 (quantitative)
Proportion achieving control of blood pressure (< 130/80 mmHg) among patients with hypertension	MOH data from DHIS2 (quantitative)
Number of patients receiving hypertension medication treatment per month (“hypertension treatment rate”)	MOH data from SIGSA (quantitative)
* Implementation outcomes*	
Adoption	Number of participating health facilities, defined as having enrolled at least one patient with hypertension or diabetes (quantitative); reasons for variation (qualitative)
Fidelity (health worker training on hypertension and diabetes treatment protocols)	Proportion of health workers in each district attending all training sessions, chart audit of prescriptions to assess guideline concordance (quantitative); reasons for variation (qualitative)
Fidelity (team-based care and task sharing)	Proportion of primary health districts conducting at least one care coordination meeting; reasons for variation (qualitative)
Fidelity (access to medicines and diagnostics)	Monthly availability of MOH medications and diagnostics (quantitative) and reasons for variation (qualitative)
Fidelity (facility-based electronic monitoring tool)	Proportion of patient visits captured in DHIS2 each month compared to comprehensive records in SIGSA (quantitative) and reasons for variation (qualitative)
Fidelity (systems monitoring and feedback)	Proportion of quarterly reports viewed by health district administrators (quantitative) and reasons for variation (qualitative)
Usability (facility-based electronic monitoring tool)	System Usability Scale [48, 49] (quantitative) and reasons for variation (qualitative)
Sustainability	Program Sustainability Assessment Tool [50, 51] and Clinical Sustainability Assessment Tool [52, 53] (select questions)
* Patient-related outcomes measures*	
Diabetes distress	Diabetes Distress Scale [54, 55], 2-item screening and physician distress subscale
Disability	WHO Disability Assessment Schedule [56]
Multimorbidity treatment burden	Multimorbidity Illness Perceptions Scale [57, 58], treatment burden subscale

*Abbreviations*: *CSAT* Clinical Sustainability Assessment Tool, *DBP* Diastolic blood pressure, *DHIS2* District Health Information System, *FBG* Fasting blood glucose, *MOH* Ministry of Health, *PSAT* Program Sustainability Assessment Tool, *RBG* Random blood glucose, *SBP* Systolic blood pressure, *SIGSA* Health Management Information System, *WHO* World Health Organization

### Study procedures

**Fig. 3:**
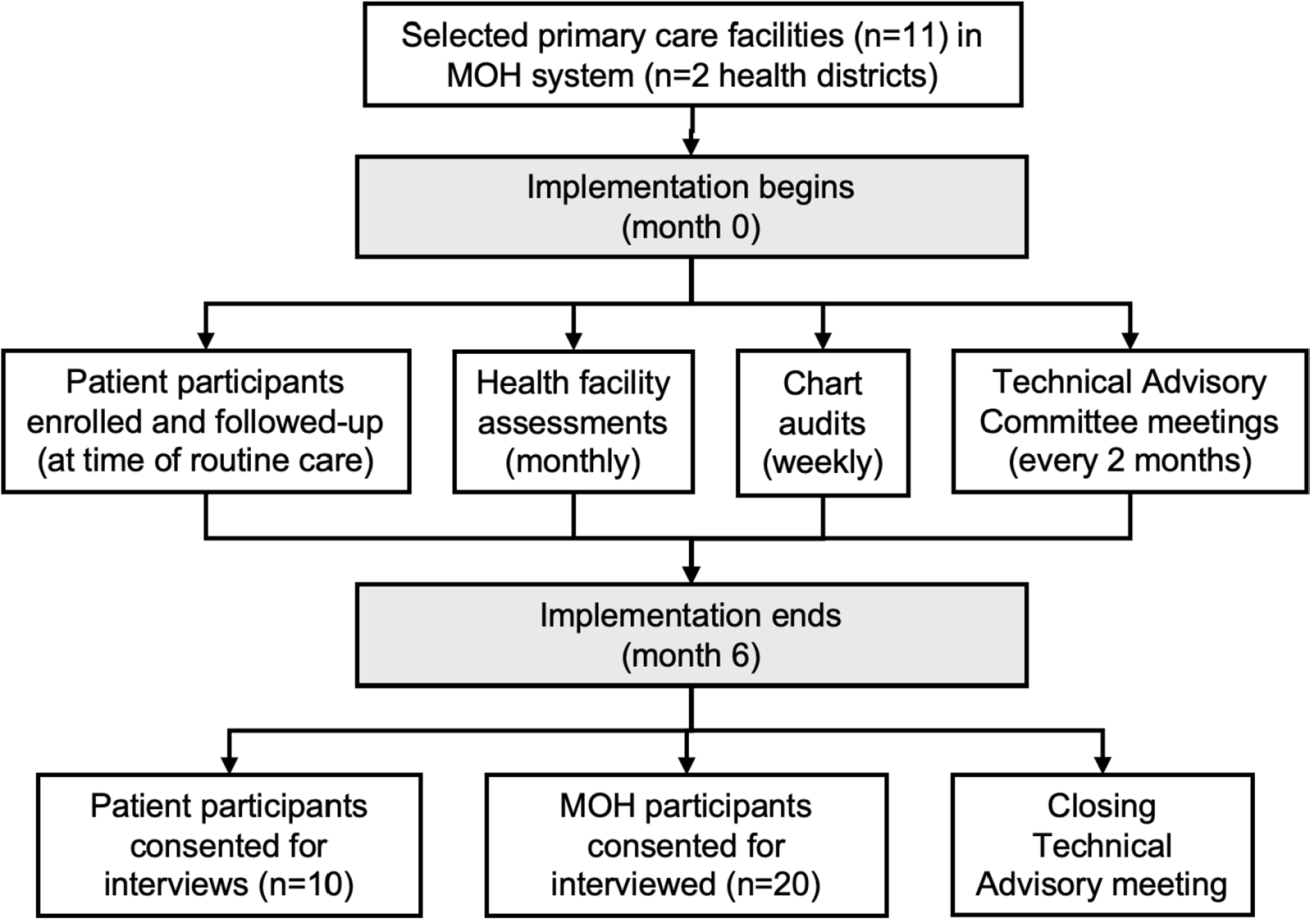
Summary of study procedures. Abbreviations: MOH, Ministry of Health

A summary of study procedures is shown in Fig. 3.

#### Recruitment

All new or existing patients with diabetes, hypertension, or both receiving care at the participating MOH primary care facilities will be enrolled in the DHIS2 system. (See “ Eligibility criteria” above.) Recruitment activities will be carried out that align with routine outreach of each MOH health facility. These activities may include meetings with local leaders, public posters, and brief announcements on social media platforms or the radio.

A subset of patient participants will be recruited for questionnaire assessments and interviews. (See “ Sampling and sample size considerations.”) Study fieldworkers who are not MOH employees will make initial contact via home visits, phone calls, or encounters at MOH health facilities. Participants who express interest in this part of the study will then receive a visit by study fieldworkers to complete informed consent and interview assessments.

All MOH participants and members of the Technical Advisory Committee will be asked to complete a structured questionnaire. The study team will use lists of personnel participating in training sessions and contacts at each health district to identify MOH participants. Members of the Technical Advisory Committee will be recruited using INCAP’s local contacts and connections.

#### Baseline visits at primary health care facilities

At baseline, study staff will visit all participating health centers and health posts to complete a baseline needs and readiness assessment based on HEARTS monitoring guidelines and the WHO Service Availability and Readiness Assessment tool [25, 44, 59]. Topics covered include population served, clinical services offered, available resources, staffing, and other topics relating to HEARTS.

#### Monthly follow-up visits at primary healthcare facilities

Study staff will conduct monthly follow-up visits at each primary healthcare facility to monitor the availability of medications and supplies, review patient registration in the DHIS2 system, assess implementation of collaborative care meetings, and provide support for any implementation issues relating to HEARTS. Study fieldworkers will also maintain a field log with notes from primary health facility visits.

#### Closing interviews with diabetes patient participants

Interviews consisting of structured and semi-structured questions lasting approximately 45 min will be carried out at the project’s termination. The focus will be on diabetes, as the study team has conducted extensive interviews with hypertension patients in prior HEARTS-aligned projects. The structured portion will cover patient-reported outcome measures, and the semi-structured portion will cover acceptability and implementation determinants (i.e., barriers and facilitators). The Tailored Implementation in Chronic Diseases (TICD) checklist will guide semi-structured interviews [60]. Visits will be carried out in the patient’s home or another convenient location. Interviews will be in Spanish or a local Mayan language, as preferred by the patient.

#### Closing interviews with MOH health workers and administrators

All MOH participants participating in the pilot will be invited to complete a structured questionnaire, focusing on feasibility (FIM instrument), sustainability (Program Sustainability Assessment Tool [50, 51] and Clinical Sustainability Assessment Tool [52, 53]), and usability of the facility-based electronic monitoring tool (System Usability Scale [48, 49]). Additionally, a subsample will participate in semi-structured interviews lasting approximately 45 min. (See “ Sampling and sample size considerations.”) The TICD checklist will guide the semi-structured interviews [60]. Interviews may be conducted in person or virtually.

#### Technical advisory committee meetings

We will establish a Technical Advisory Committee to provide high-level coordination among national and subnational authorities, as recommended in the HEARTS Implementation Guide [25]. The Technical Advisory Committee will play a critical role to provide guidance during the pilot and to plan for future scale-up. Members will likely include MOH administrators at the national, departmental, and health district levels, physicians and professional nurses working in each health district, representatives of the Guatemalan PAHO office, and other stakeholders (10–15 total members). Study staff at INCAP will organize meetings every 2 months during the trial and a posttrial closing meeting. Meetings will be conducted virtually and will be recorded for members who cannot attend a given session. Written meeting notes also will be shared after each session.

During the meetings, study staff will present project updates for open discussion. Using an implementation mapping approach [61], members then will discuss the implementation determinants (i.e., barriers and facilitators) that emerge during the trial for each intervention component, select implementation strategies to address component-specific determinants, clarify the causal mechanisms through which implementation strategies operate, and provide feedback on a consolidated implementation package. Study staff will guide discussions of implementation strategies using different structured tools. The Expert Recommendations for Implementing Change (ERIC) compilation and prior mappings of ERIC to lower-middle-income countries will be used as a foundation for proposed implementation strategies [62, 63]. Guidance from Proctor et al. will be used to specify implementation strategies [64]. The APEASE (acceptability, feasibility, effectiveness, cost-effectiveness, side effects or unintended consequences, safety, and equity) tool will be used to prioritize implementation strategies [65]. Finally, causal pathway models will be presented to link implementation strategies, mechanisms, and key implementation outcomes [66, 67].

#### Chart audits

Each week, a data manager will review new data entered into the DHIS2 system for missingness and errors. Physicians on the study team also will perform a clinical audit of at least 25% of patient visits. The physicians will use a structured checklist to rate the guideline concordance of clinical care and quality of data entry.

### Data collection and management

Data will be collected using different collection methods. Clinical data from patient participants will be entered into DHIS2 by MOH health workers who provide standard clinical care during routine visits. DHIS2 data are stored on INCAP’s server, as approved by the MOH. Data from structured assessments will be collected electronically using a cloud-based version of REDCap hosted at INCAP. Structured assessments include health facility monitoring, chart audits, and questionnaire data from closing interviews with patients and MOH staff. Data entry and quality control checks will be performed by study staff on all structured data entered into REDCap. Qualitative data from semi-structured interviews will be collected in the field by a trained qualitative researcher on the study team. Other qualitative data will include field notes, meeting notes, and study team reflections on implementation progress and challenges [68]. Qualitative data will be securely stored on the University of Michigan’s institutional Dropbox account with routine backups to an encrypted hard drive.

### Sampling and sample size considerations

The sample of health facilities will include 9 health posts and 2 health centers for a total of 11 primary health facilities. No formal sample size calculation was performed [69, 70]. This sample of health facilities and their catchment area population are consistent with recommendations in the HEARTS Implementation Guide [25].

The planned sample of patient participants will be approximately 100 individuals or 50 participants per health district. Based on the study team’s prior experience, the anticipated total breakdown is *n* = 45 patients with hypertension only, *n* = 45 with diabetes only, and *n* = 10 patients with both hypertension and diabetes. However, the improved clinical services may attract a greater number of patients to MOH care than anticipated for the pilot. Of these, a subsample of 10 participants with low versus high retention levels (defined by number of clinical visits within the study period; 5 participants per group) will be purposively selected among groups of individuals who had enrolled in the first two months of the study. To improve understanding of how diabetes can be integrated into the HEARTS hypertension primary care model, we will purposely sample patient participants with diabetes.

The anticipated sample of MOH participants working to implement HEARTS will be approximately 50 participants. Of these, a subsample of 20 will be purposively selected for semi-structured interviews based on high versus low perceptions of intervention feasibility and MOH role (i.e., physicians or physicians-in-training, professional nurses, auxiliary nurses, and administrators).

Including patient and MOH participants, a total of 30 semi-structured interviews are planned to achieve thematic saturation [71, 72]. Interviews will be analyzed as they are conducted, and more may be added if thematic saturation is not achieved.

### Analysis plan

#### Quantitative analysis plan

Clinical data will be analyzed using descriptive statistics and multilevel regression models of individual-level data adjusting for clustering of participants within primary health facilities. Sociodemographic variables such as age, sex, education level, and other characteristics may be explored in regression models if sample sizes permit. Stata will be used for quantitative analyses.

#### Qualitative analysis plan

Semi-structured interviews will be recorded and analyzed in Spanish using qualitative directed-content analysis [73]. We will only transcribe recordings for interviews undertaken in a local Mayan language. In these cases, professional linguists will translate and transcribe into Spanish for analysis. Constructs from the Tailored Implementation in Chronic Diseases checklist will guide qualitative coding [60]. Two members of the research team proficient in Spanish will independently code transcripts, and the principal investigator will reconcile differences. Dedoose will be used for qualitative analyses [74].

#### Mixed-methods analysis plan

**Fig. 4:**
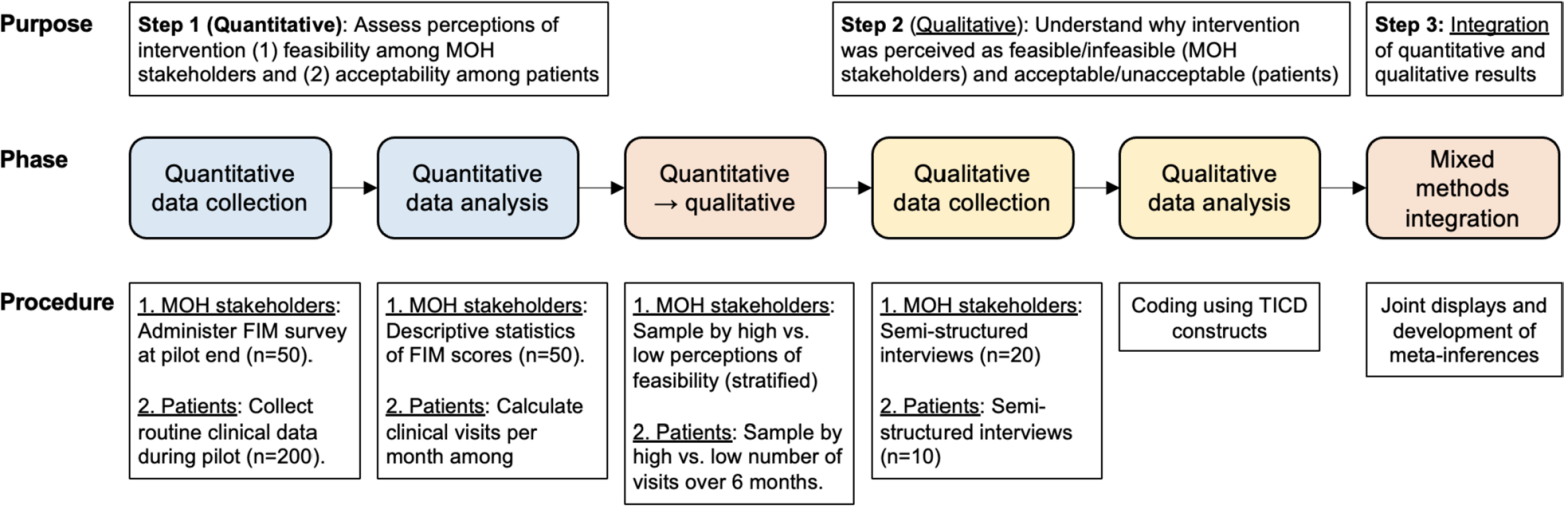
Explanatory sequential mixed-methods procedural diagram. Abbreviations: FIM, Feasibility of Intervention Measure; MOH, Ministry of Health; TICD, Tailored Implementation in Chronic Diseases

The mixed-method analysis will be based on the explanatory sequential design, as depicted in Fig. 4. Quantitative and qualitative findings of primary outcomes will be integrated using joint displays, which are a mixed methods visual technique [75]. Joint displays will show quantitative data next to illuminating participant quotes. Analysis of integrated quantitative and qualitative data will permit the study team to draw meta-inferences regarding the projects’ feasibility and to facilitate future implementation planning.

### Data and safety monitoring plan

#### Adverse event reporting

The intervention in this study is focused on improving standard-of-care treatment of diabetes or hypertension delivered by the MOH health workers in MOH facilities. Therefore, MOH staff will be responsible for providing care for patient participants who experience an adverse event such as hypotension, hypoglycemia, or other known adverse drug effects. In our reporting role, the study team will review MOH records for adverse events (including those related to common medications), unanticipated problems, and other reportable information.

#### Monitoring the study

The study team will conduct monthly scheduled assessments of study recruitment, data integrity and quality, adverse events, withdrawals, and compliance with protocol. No interim analyses are planned. The trial will not employ stopping rules nor a Data Safety Monitoring Board because the study carries no more than minimal risk to participants. If the study investigators and Technical Advisory Committee think there would be potential benefit in extending the pilot trial, and funding is available, then an extension may be considered (e.g., from 6 to 12 months in duration).

### Posttrial care

This trial is embedded in standard MOH primary care. All patient participants will be able to continue receiving diabetes or hypertension care according to national standards after the study closes. Depending on patient volume and logistics in the MOH, some health posts participating in the trial may refer patients to health centers for continuation of care.

### Dissemination of results

Project results will be shared through a structured dissemination strategy that includes timely registration and reporting on ClinicalTrials.gov, meetings in each MOH health district at the end of the project, presentations to the Technical Advisory Committee, nontechnical reports in Spanish and English disseminated through established noncommunicable disease research and policy networks in which INCAP participates, and academic research conference presentations and peer-reviewed journal publications. Open-access journals will be prioritized for publication, and eligibility for authorship on academic products will be guided by the International Committee of Medical Journal Editors guidelines.

## Discussion

This pilot study will address the critical need for generalizable knowledge on how to close the “implementation gap” for CVD prevention in primary care in low- and middle-income countries. To our knowledge, the study will be among the first to investigate how integrated hypertension and diabetes management based on HEARTS can be implemented in a national primary care health system [10]. The investigators and local stakeholders in Guatemala will use results to plan a subsequent hybrid type 2 or type 3 effectiveness-implementation trial throughout Guatemala.

A key objective of the study pilot also is to explore HEARTS-aligned implementation strategies using the structured implementation mapping approach with high-level stakeholders in Guatemala. Causal pathway models also will be developed to connect implementation strategies, mechanisms, and key implementation outcomes. The implementation strategies developed in this study can inform future HEARTS projects in Guatemala and other low- and middle-income countries.

There are a few limitations and potential problems in this pilot study. First, while not designed nor powered to make causal estimates of clinical or implementation changes, the study’s sample size of health facilities and participants will be adequate for assessing the primary outcomes of feasibility and acceptability. Second, this study is embedded in the MOH primary care system. Potential problems include political crises, health worker strikes, changes in MOH leadership, and other unexpected events. To address these challenges, we will engage the Technical Advisory Committee, including high-level national authorities, and leverage INCAP’s unique position as a public institution with longstanding government connections and buy-in. Third, implementing the DHIS2 electronic monitoring tool will be ambitious given that many MOH health facilities are in isolated rural areas with unreliable Internet connectivity. If it is not possible to implement the DHIS2, we will try to leverage the existing workflows of the Health Management Information System (SIGSA) for the study.

### Supplementary Information

**Additional file 1.** SPIRIT 2013 Checklist: Recommended items to address in a clinical trial protocol and related documents*.

**Additional file 2.** CONSORT 2010 checklist of information to include when reporting a pilot or feasibility trial*

**Additional file 3.** The TIDieR (Template for Intervention Description and Replication) Checklist.

## Data Availability

This project will produce multiple types of data, including patients’ clinical information, health facility assessments, and structured and semi-structured interviews. Deidentified data, analytic code, and data dictionaries will be made available on the NHLBI BioLINCC data repository (https://biolincc.nhlbi.nih.gov/) after the study concludes. Semi-structured interview transcripts and structured questionnaire data will not be shared due to privacy concerns and risk of re-identification.

## Abbreviations

AIM: Acceptability of Intervention Measure
APEASE: Acceptability, feasibility, effectiveness, cost-effectiveness, side effects or unintended consequences, safety, and equity
DBP: Diastolic blood pressure
DHIS2: District Health Information System 2
FBG: Fasting blood glucose
FIM: Feasibility of intervention measure
HbA1c: Hemoglobin A1c
HEARTS: Healthy lifestyle counseling, evidence-based protocols, access to medicines, risk-based management, team care and task sharing, and systems monitoring
INCAP: Institute of Nutrition of Central America and Panama
MOH: Ministry of Health
PAHO: Pan American Health Organization
PROEDUSA: Department of Health Training and Education
PSAT: Program Sustainability Assessment Tool
SBP: Systolic blood pressure
SIGSA: Health management information system
TICD: Tailored Implementation in Chronic Diseases
WHO: World Health Organization
